# Supplementary material for: Global remapping of the sensory homunculus emerges early in childhood development
Source: Nat Commun. 2026 Feb 24;17:1591. doi: 10.1038/s41467-025-66539-5 (PMC12932738; doi:10.1038/s41467-025-66539-5)
Supplement: Supplementary file 1 — Supplementary Information [file 41467_2025_66539_MOESM1_ESM.pdf]

Supplementary Methods

Co-use formula

The co-use measure used for our behavioural analysis (Figure 1A) is an estimated index that reflects both the number of body parts used and how consistently they were used, with higher values indicating more usage or more consistent usage. The function is the sum of two components: C1 + C2. Component C1 (equation 1) counts the number of body parts that were used during the observation period (an integer value). It is calculated by counting any body part with a usage proportion greater than zero.

$$C1 = \sum_{i=1}^{n} U_i \tag{1}$$

U_i is a binary indicator variable for the ith body part which is equal to one if the ith body part was used ($p_i>0$) or zero if it was not used ($p_i=0$), and n is the total number of body parts considered. Let's indicate with p_i the proportion of use of the ith body part. Component C2 (equation 2) adjusts the index based on the consistency of usage across the used body parts. It is computed as the average of the difference between each used body part and its proportion.

$$C2 = \frac{\sum_{i=1}^{n}(U_i - p_i)}{\sum_{i=1}^{n} U_i} \tag{2}$$

The value of C2 is either **zero** or **negative**. It's zero if all body parts were used for the entire task duration. However, it becomes negative if at least one body part was used for less than the total task time. Essentially, C2 accounts for differences in how long each body part was engaged.

A smaller penalty from C2 (meaning a value closer to zero) results in a higher overall co-use index. This happens when the body parts used have similar or high proportions of engagement time. Conversely, if body part usage is highly uneven—for example, one body part is used extensively while others are barely involved—C2 will significantly decrease the co-use index. This reflects a less cooperative or balanced usage of body parts.

The final equation to estimate the co-use index is indicated in equation 3:

$$\text{co-use} = C1 + C2 \tag{3}$$

Supplementary Results

Activity analysis

To complement the peak position analysis, we also analysed activity levels at the peak of each individual participant's body part representations along S1. Specifically, when comparing individual Z values for peak activity we identify significant main effects of limb difference for the closer neighbours of the deprived hand – the residual arm and the lower face showing stronger activity in CLD individuals (arm: $F_{1, 226.50} = 71.70$, $p < 0.001$; lower face: $F_{1, 226.77} = 14.32$, $p < 0.001$), but not in further body parts (feet, legs and torso; all $p > 0.179$). This indicates that, while deprivation impact the distribution of the body parts along the extent of the homunculus, activity levels are affected more locally. Interestingly, the closest neighbour to the deprived hand area—the upper face—did not show a main effect of limb difference ($F_{1, 276.31} = 2.33$, $p = 0.128$). This result, which replicated a similar effect[1], indicates that the increased activity found for the deprived hand's other neighbours may not be independent of use.

Age effects were mostly anecdotal (a main effect for the lower face: $F_{1, 226.77} = 7.62$, $p = 0.006$ and the feet: $F_{1, 213.11} = 12.03$, $p = 0.001$); a weak trend towards a main effect of age for arm ($F_{1, 226.50} = 2.84$, $p = 0.093$, was further confirmed by a Bayes analysis: $BF_{10} = 8.18e^6$). Interactions between age and limb difference were also sparce, and only conclusively apparent with Bayes statistics (arm: $F_{1, 226.50} = 3.11$, $p = 0.079$, $BF_{10} = 5.84e^6$; feet: $F_{1,213.11} = 3.72$, $p = 0.055$, $BF_{10} = 83.52$). This spatially intermitted pattern of results diverges from the more localised effects found for deprivation, and indicates that S1 activity and remapping may further scale with age, providing further evidence for the opportunity for the features of the body maps to be modulated later in life. Nevertheless, as activity levels are more dependent on the stimulation paradigm and strength (in comparison to peak location), we did not follow up on this initial result. For the complete results of the omnibus ANOVA (across the 5 main body parts) see Supplementary table 9.

Supportive analysis for the impact of behaviour on topographic shift

To further validate our correlation analysis, we conducted an additional permutation test. This time, our compensatory behaviour measure was based on the relative frequency each body part was used across all sub-tasks (e.g., if the residual arm was used in 13 out of 15 sub-tasks, the index was 0.867). Similar to our original approach, we analysed deviations in activity profiles and behavioural performance for each CLD individual relative to their peer control group. We observed very consistent results: The averaged Spearman correlation was 0.52 ($p < 0.001$) for children and 0.27 ($p = 0.003$) for adults. Moreover, children's mean correlation remained significantly larger than adults' ($t=3.12$, $p=0.002$). These findings strongly corroborate our original observations: individual behavioural adaptations are indeed reflected in variations in S1 brain topography, particularly during early development.

To further validate our key result, we conducted a Leave-One-Subject-Out (LOSO) validation. For each group, we iteratively removed one participant and then recalculated the mean correlation for the remaining individuals. This process created a distribution of "leave-one-out" mean correlations for each group. The LOSO validation confirmed high consistency in our group-averaged correlations. For the CLD children, the mean of these LOSO correlations was 0.593 (standard deviation = 0.0156, range = 0.0571), perfectly matching their original group mean of 0.593. Similarly, for the CLD adults, the LOSO mean was 0.393 (standard deviation = 0.0215, range = 0.0714), consistent with their original mean of 0.393. The small standard deviations and narrow ranges observed within both groups indicate that our reported average correlations are robust and not disproportionately affected by any single participant.

Computational Model

In this section, we first outline how the model's initial connection weights were set, then present a parameter-sensitivity analysis, a method used to assess how variations in a model's input parameters influence its output[2–4]. We use this approach to explore the role of behaviour in homunculus adaptation and to test the model's robustness to parameter changes. To further examine the generality of our findings, we introduce a rudimentary scenario of the homeostatic model. Finally, we examine how varying the relative stimulation frequency of the hand impacts the model's ability to satisfy key empirical observations.

Setting of Initial Connection Weights

Connection Weight Initialization Pattern

Initial connection weights between each body part and internal neurons follow a bell-shaped function (see Figures S9 and S12). The rationale behind this setting is described in the subsection Initial weights of the model of main Methods section. The weight between a body part BP and an internal neuron j, for all body parts except the hand (i.e., Palm and Fingers), is calculated as follows:

$$W_{j,BP} = e^{-\frac{1}{2}\left(\frac{(j-1)-c_{BP}}{\sigma_{BP}}\right)^2} + C$$

where $j \in \{1,2,\dots,N\}$, with $N = 50$ is the number of internal neurons; c_{BP} denotes the centre of the Gaussian curve connection pattern associated with body part BP , i.e., the neuron position on the S1 strip with the strongest connection to BP ; σ_{BP} determines the width of the Gaussian and $C = 0.05$ is a vertical offset that ensures weak connections to all internal neurons.

For body parts $BP \in \{Palm,\ Fingers\}$, the Gaussian weights are multiplied by a factor $F_{Palm,Fingers} \geq 1$, so the weights take the following form:

$$W_{j,BP} = F_{Palm,Fingers} \cdot e^{-\frac{1}{2}\left(\frac{(j-1)-c_{BP}}{\sigma_{BP}}\right)^2} + C.$$

This enables stronger connections between these body parts and internal neurons. In contrast, for CLD body parts $BP \in \{Palm\ nerves,\ Fingers\ nerves\}$, connections are weakened by a peripheral reorganisation factor $F_{Periph\ reorg} \leq 1$

$$W_{j,BP} = F_{Periph\ reorg} \cdot \left(F_{Palm,Fingers} \cdot e^{-\frac{1}{2}\left(\frac{(j-1)-c_{BP}}{\sigma_{BP}}\right)^2} + C\right).$$

Model parameters

For all models, the centres of the bell-shaped initial connection patterns for individual body parts are fixed at:

$$[c_{Genitals},\ c_{Foot},\ c_{Leg},\ c_{Torso},\ c_{Arm},\ c_{Palm},\ c_{Fingers},\ c_{Upper\ Face},\ c_{Lower\ Face},\ c_{Throat}] =$$

$$= [12.5,\ 15.17,\ 17.83,\ 20.5,\ 23.17,\ 25.83,\ 28.5,\ 31.17,\ 33.83,\ 36.5].$$

Corresponding to (genitals, foot, leg, torso, arm, palm, fingers, upper face, lower face, throat). The remaining parameters are listed in the following table:

Parameter	Main model (Figure S9)	Rudimentary model (Figure S12)
σ_{BP} for all body parts BP except for Palm, Fingers and their corresponding nerves	4	4
σ_{Palm}, $\sigma_{Palm\ nerves}$, $\sigma_{Fingers}$, σ_{F}[illegible]	5	4
$F_{Palm,Fingers}$	5	1
$F_{Periph\ reorg}$	0.15	0

Role of behaviour for homunculus adaptation

In the Results section of the main article, we used the same *stimulation probabilities* for both the CTR and CLD participants, meaning that behavioural differences between the groups were not required to explain the observations using the homeostatic model. Here, we examine the potential influence of behavioural differences between the two groups.

The exact stimulation probabilities for individual body parts, which would encompass the full range of human activities (e.g., sleeping, walking, etc.), are not well established, even for the CTR group. Since the main differences in body part usage between CLD and CTR participants likely occur during manipulation tasks, we used our behavioural data to identify which body parts are used significantly more or less by the CLD group compared to CTR participants. Our data show that the CLD group uses the leg, torso, and lower face more frequently, while the arm is used less (see Figure 1B). However, because our behavioural data mainly reflect manipulation tasks, these values are not directly applicable to the homeostatic model, which also needs to account for non-manipulative activities. Therefore, we tested the model under different sets of body part usage probabilities to capture a broader range of activities.

We hypothesised that increased stimulation of certain body parts in CLD individuals could counteract or even reverse the effects of hand stimulus deprivation, particularly in areas closely linked to the more frequently stimulated parts. For example, before homeostatic adaptation, if hand deprivation reduces a neuron's mean activity by -0.5, this reduction triggers an increase in synaptic weights once homeostasis is applied. Now, in a different scenario where another body part is more often stimulated, resulting in a +0.7 increase in mean neural activity, the combined effect on the neuron would be +0.2 (0.7 - 0.5). In this case, homeostatic adaptation would lower synaptic weights to restore the neuron's activity to its baseline level. This adjustment would be in the opposite direction of the changes seen with hand deprivation alone. In summary, we predict that increased use of certain body parts in CLD individuals could mitigate or even reverse cortical changes linked to hand stimulus deprivation.

To qualitatively analyse the impact of more frequent leg use in CLD individuals, we performed a parameter sensitivity analysis by adjusting the stimulation probability for the leg in the CLD group while keeping the CTR group's probabilities unchanged from the baseline. Specifically, we increased the leg's stimulation probability by 25%, 150%, and 300%, with all other model parameters remaining as in the baseline scenario. Figure S10 compares the original model with these modified versions.

The model produced two distinct outcomes based on the level of leg stimulation increase. With a 25% increase (i.e., 125% of the baseline), none of the key observations were violated (see Figure S10, Panel B). However, with a 150% or 300% increase, observations 2 and 3 were violated. Notably, at a 300% increase, the magnitude of the peaks for the foot and leg were higher for CTR participants, deviating from the expected pattern (see Figure S10, Panel D). Additionally, the expected peak position for foot activity was also disrupted.

Similarly, we examined how the homunculus adapts when there is a decrease in arm and hand nerves stimulation in CLD individuals. We reduced the original stimulation probability by 15% (which corresponds to the decrease observed in our behavioural data during manipulation tasks) and by 30%. Since it is unlikely that overall daily activity would decrease by more than double the observed reduction during manipulation tasks, we did not explore reductions beyond 30%. Figure S11 presents a comparison of all conditions for arm and hand nerve stimulation.

In the lower stimulation scenario, the peak magnitudes for CLD participants were higher, reinforcing Observations 1 and 3, with none of the key observations being violated. Additionally, for CLD participants, there were slight shifts in the activation peaks for arm (lateral direction) and lower face (medial direction), consistent with Observations 1 and 2. This indicates that reduced limb-related stimulation due to behavioural changes in CLD participants has effects similar to reductions caused by limb difference alone.

In summary, guided by behavioural data, we systematically investigated the effects of increased leg stimulation and decreased upper limb stimulation in CLD individuals. For the leg, a 25% increase did not violate any of the four key findings but increases above 150% violated Observations 2 and 3. The results suggest that the empirically observed higher body part usage in CLDs does not generally contribute to the crucial observed activity differences between both groups. Instead, it appears to reduce these differences. However, these differences remain present unless the behavioural differences exceed a certain threshold. For reduced upper limb stimulation, decreases of up to 30% did not violate any of the four key observations. The decrease even strengthens some of the activity differences between both groups suggesting that it has a similar effect on the activity patterns as the input reduction caused by limb difference alone.

Rudimentary scenario homeostatic model

To demonstrate the robustness of our model results to variations in parameter choices, we present a simplified, or "rudimentary," experimental scenario. Unlike the base model, this version assumes no input from the palm and finger nerves in CLD individuals, meaning there is no peripheral reorganization. Additionally, for both CLD and CTR groups, all body parts—including the palm and fingers—are assigned identical stimulation probabilities (P_{BP}=0.05 for all body parts), and the same bell-shaped initial connection patterns between body parts and the homunculus strip are used (see Figure S12).

Figure S13 shows node activity during tactile stimulation of individual body parts. Remarkably, the model replicates most of the key empirical findings (see Results section): 1) The arm activation peak in the CLD group is higher and shifted to the right compared to the CTR group; 2) except for the foot, all CLD activation peaks are shifted toward the centre of the strip relative to CTR peaks; 3) all activation peaks in the CLD group are higher than those in the CTR group. However, in this simplified scenario, empirical property 4—where there is a decrease in activation

peak magnitude from the foot to the leg and torso, and an increase from the upper face to the lower face—is only reproduced in the CTR group, not in the CLD group.

In summary, even in this rudimentary scenario, the model replicates most of the key empirical findings, highlighting the robustness of our results. However, to fully capture all the empirical observations, a more complex model is required—one that incorporates peripheral reorganization, higher hand (or corresponding nerve) stimulation frequencies, and stronger initial connections between the CTR hand and the cortical strip.

Justification of Stimulation Probability Settings

Here, we systematically analyse how varying the relative stimulation frequency of the hand (i.e., palm and fingers or the corresponding nerves) influences the model's ability to satisfy key Observations.

Since no comprehensive empirical data exist on estimated stimulation frequencies of individual body parts across all daily activities, we adopted a parsimonious approach: all body parts share the same probability of stimulation, except for the hand, which was assigned a higher probability, due to its role in tool and object manipulation.

When the ratio of hand to other body parts' stimulation probabilities is fixed, the specific probability values themselves are less critical. This is because the multiplication of probabilities merely scales the activations after homeostasis; therefore, it does not influence how the model satisfies the key Observations. Such scaling can be counterbalanced by adjusting the homeostatic Mean Neural Activity. For example, doubling all stimulation probabilities yields equivalent activations if the homeostatic Mean Neural Activity of all neurons is also doubled.

To explore the effect of hand stimulation probability, we performed an analysis where the hand was stimulated 1, 3, and 5 times more frequently than other body parts. Specifically, the tested hand probabilities were 0.05 (equal to other body parts, Figure S15A), 0.15 (three times higher, used in the main model, Figure S15B), and 0.25 (five times higher, Figure S15C). The results indicate that lower hand stimulation probabilities lead to an increase in the arm activation peak, whereas higher hand stimulation probabilities result in a decrease of the arm activation peak. For a hand probability of 0.05, only Observation 2 was violated for the foot, while all other Observations remained preserved.

Overall, these findings indicate that the model is highly robust to reasonable changes in hand-stimulation probability. A value of 0.15 was selected for the main model because, based on visual comparison, the relative size of the CLD Arm peak (compared to other peaks) most closely resembled the corresponding relative peak sizes observed in empirical data.

Topographical changes beyond S1

We also examined activity changes in secondary somatosensory cortex (S2), to determine if the changes found in S1 are cascaded downstream. We repeated the same set of analyses reported in the S1 hand area (see Results section in the main text). When looking at activity levels across body parts and groups (Figure S14), we observe a significant interaction effect between age and body part ($F_{4,248}$ =7.08, p<0.001), which was driven by the arm and chin (arm: $F_{1,135.17}$=4.28, p= 0.040; chin: $F_{1,135.17}$=8.82, p=0.004). We found a trending significant main effect of limb difference

for the chin only ($F_{1,135.17}$=3.68, p= 0.057), which remained ambiguous following Bayes analysis (BF_{10}=0.97), and no significant interactions with limb difference, both for the omnibus model or the follow up ANOVAs per body part (all p > 0.101). When considering representational dissimilarity, we again find no effects of limb difference (all p>0.177) and we observed a significant main effect of age ($F_{1,62}$=5.18, p= 0.026) as adults showed larger distances than children.

To further confirm and assess the representational similarity in S2, we also performed a correlation analysis between the pairwise distance values in the S1 deprived hand area and in S2 for each participant. All groups showed a significant correlation between the two ROIs as indicated by one-sample t-tests against zero (all p<0.046), confirming that the representational structure is overall shared across ROIs. We observed no main effect of limb difference ($F_{1,63}$ = 0.07, p = 0.792), but we observed a significant main effect of age ($F_{1,63}$ = 6.28, p = 0.015) and a weak (and ambiguous) trend towards an interaction ($F_{1,63}$ = 2.951, p = 0.093, BF_{10}=0.92), which was driven by larger correlations in control adults relative to control children (t_{63}=3.03, p=0.004). Together, these findings indicate that the differential activity observed along S1 to express a limb difference may not be read out by S2, though null results should be interpreted with caution.

This pattern of results complements, yet clearly diverges from, the robust deprivation-related remapping observed in S1. Across all groups, pair-wise representational dissimilarity values in S2 correlated significantly with those in the deprived-hand area of S1, confirming that broad somatotopic motifs are captured in our S2 ROI. While we found some evidence to support that S2 continues to refine across development (see [5] for related results), neither activity levels nor representational distances in S2 were modulated by limb difference. This dissociation is not unexpected given the different functional roles of the two areas. Compared with S1, S2 representations are less spatially segregated[6] and are more susceptible to task demands, including object-centred features[7] and multisensory integration[8]. Our abstract, body-part-specific stimulation paradigm may therefore have lacked the ethological richness required to expose functional plasticity in S2. Together with the inconclusive Bayes Factor values, which do not provide sufficient evidence to confirm the null hypothesis, the absence of a clear remapping signature in S2 should be interpreted cautiously.

Supplementary Figures

Figure S1: Proportion of time analysis across sub-tasks.

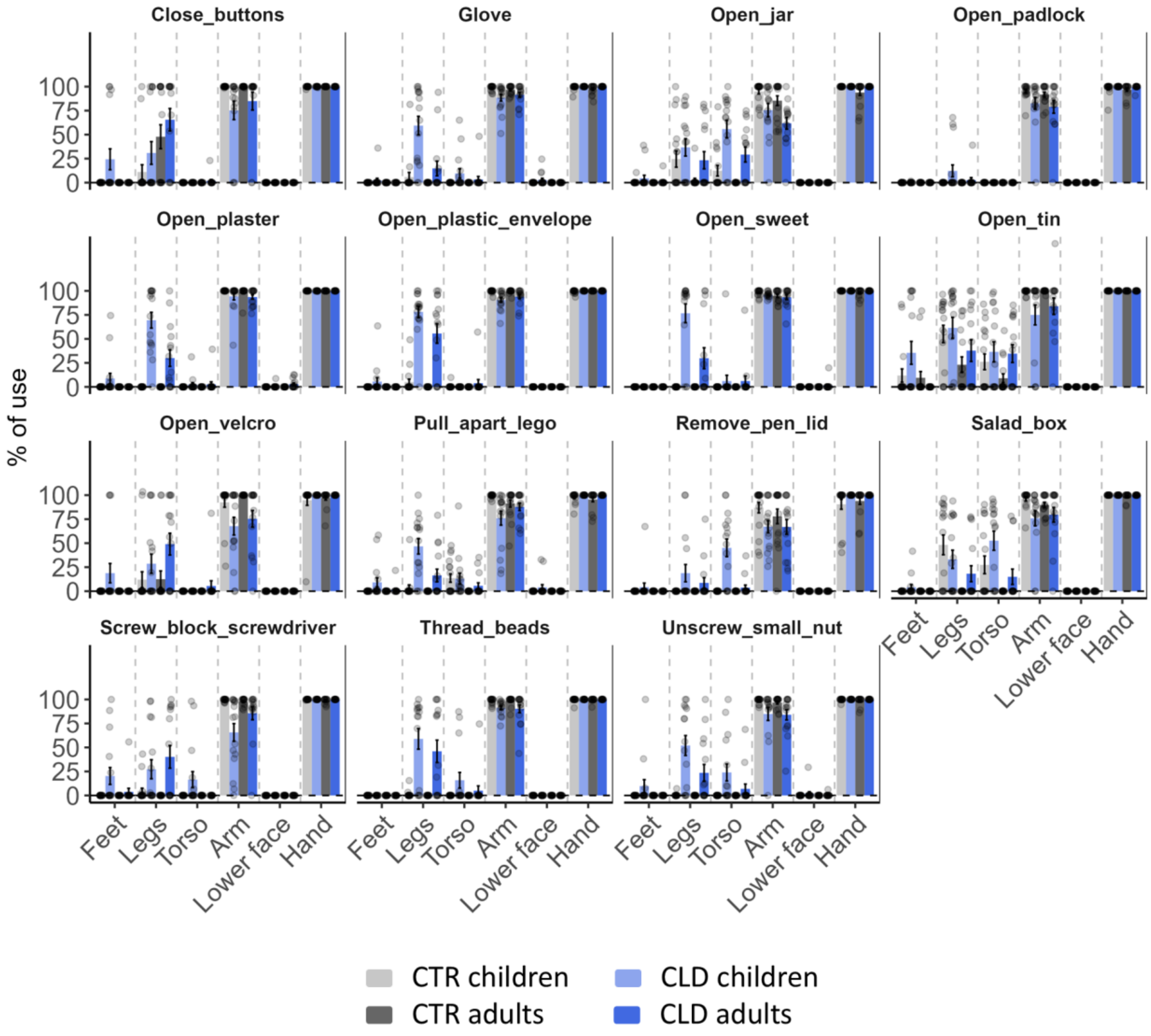

Figure S1. **Proportion of time each body part was used relative to the total task time for each task.** Performance data for each of the 15 tasks used to index compensatory behaviour (for the complete list of the tasks used, see Supplementary table 1). Individuals in the CLD group (represented by blue bars), and children in particular (light blue) tend to rely on alternative body parts to compensate for impaired function across a range of tasks that simulate everyday activities. For example, when performing tasks such as putting on a glove, individuals with CLD, especially children, often use their legs. In tasks requiring greater power grip, such as opening a salad box or a tin, the compensatory actions become even more variable. Here, individuals in the CLD group frequently utilise their feet, legs, and torso, with children exhibiting the most diverse range of adaptations. These findings underscore the flexibility and adaptability of compensatory strategies in CLD individuals, with children showing a heightened reliance on alternative body parts during task performance. *Error bars indicate standard error of the mean. CLD children (N=16), CLD adults (N=15), CRT children (N=17), CRT adults (N=16). Source data are provided as a Source Data file.*

Figure S2: Absolute time analysis for behavioural task.

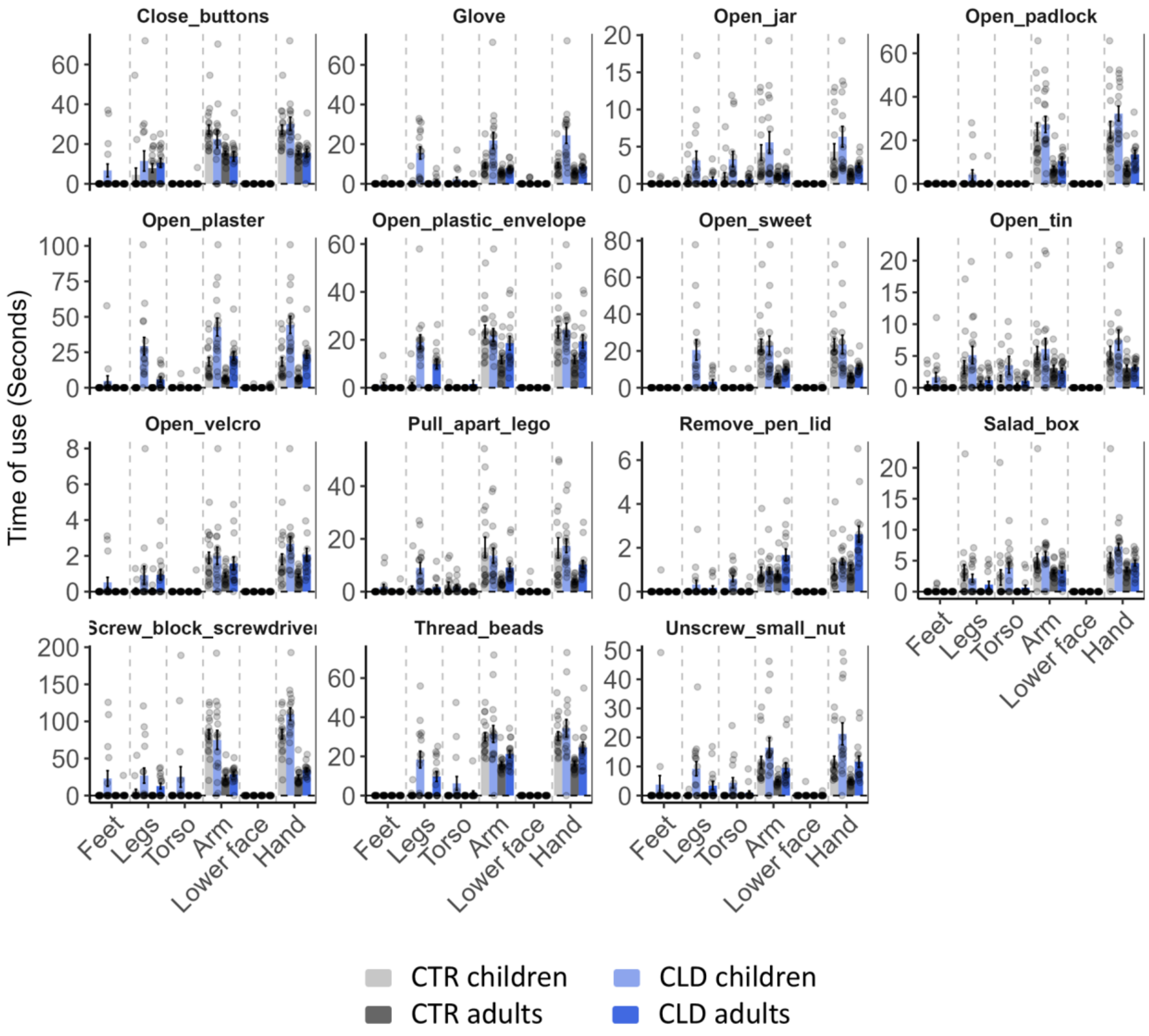

***Figure S2*. Absolute time in seconds, during which each body part was used in each task.** The time taken to complete the various tasks differed, reflecting the varying levels of task complexity. Adults (represented by darker-coloured bars), with no particular differences between CLD group (dark blue) and the CRT group (dark grey), generally completed the tasks more quickly than children, suggesting that the dexterity required for these tasks is not fully developed in younger participants. This difference in performance is most pronounced in the 'Screwdriver' task (see Supplementary table 1), which emerged as the most challenging task for children. *Error bars indicate standard error of the mean. CLD children (N=16), CLD adults (N=15), CRT children (N=17), CRT adults (N=16). Source data are provided as a Source Data file.*

Figure S3: Global S1 ROI

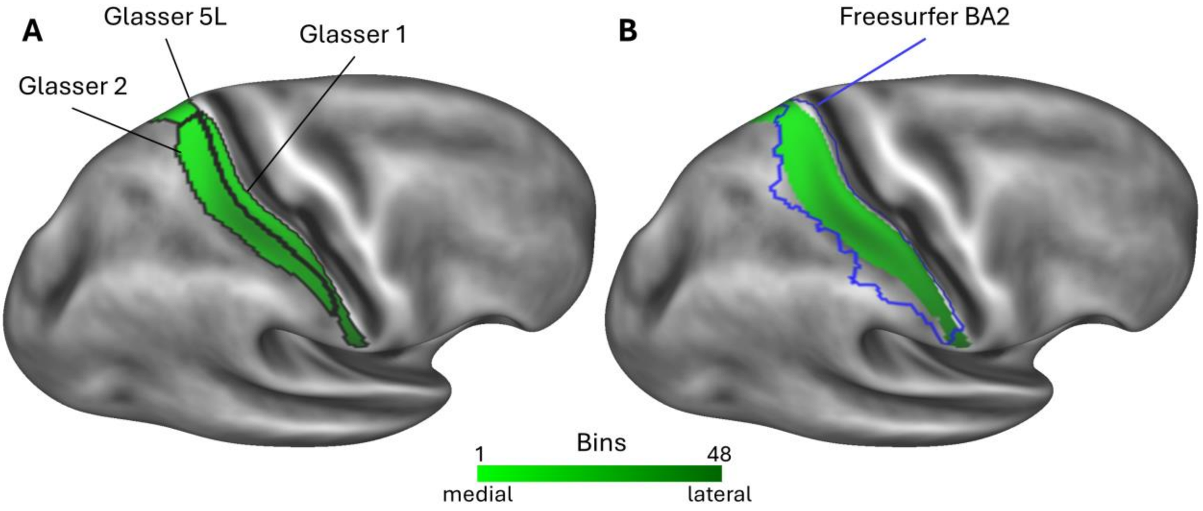

Figure S3. S1 ROI along the post-central gyrus. *To investigate global changes across all body parts along the entire postcentral gyrus, we defined an ROI in each hemisphere, visualised using gradients of green in the plots. This ROI, which included the Glasser's labels 1, 2, and 5L[9], was mapped onto the standard flat maps (FS_LR 32k) of the Human Connectome Project (HCP). The right hemisphere is displayed as an example. The shaded gradients of green represent the number of bins and their orientation along the medial-to-lateral axis. Panel A illustrates the bins-ROI along with the outlines of the labels from Glasser's atlas[9], which were used to delineate the ROI. For comparison, Panel B provides the un-thresholded outline of the FreeSurfer BA2 label.*

Figure S4: Activation maps

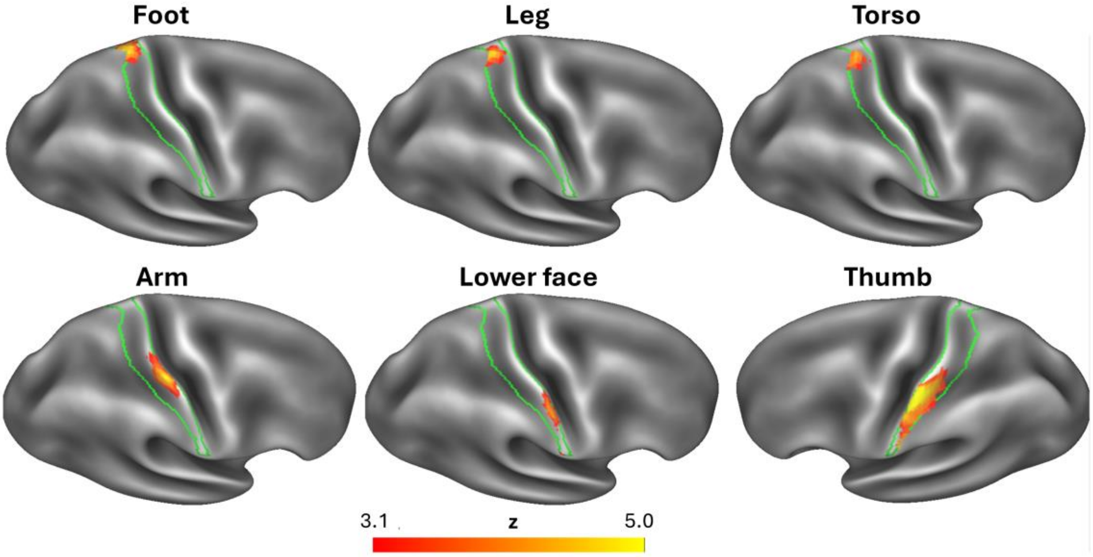

Figure S4. Activity maps when the different body parts are stimulated*. Group analysis, comprising all study participants, shows the activity patterns (z values) for each body part, reflecting the typical topographic organization (see the classical S1 topography in Figure 4 of the main manuscript) and along the higher-order sections of the somatosensory BA1-2 strip (highlighted in light green). As expected, lower body parts (Foot, Leg, Torso) elicited activity in the medial section of S1, while arm-related activity was observed in a more central region of S1. The upper body, specifically the lower face, activated the most lateral part of S1. All these activities were recorded in the hemisphere ipsilateral to the missing or non-dominant hand, corresponding to the deprived/dominant hemisphere. Stimulation of the thumb of the intact/dominant hand (in CLD/CTR participants, respectively) generated localised activity in the contralateral, intact/dominant hemisphere, as expected, in a well-established location (please note that the thumb establishes the lateral boundary of the hand representation). These findings support the consistent somatotopic organization of S1. FMRI data processing was carried out using FEAT (FMRI Expert Analysis Tool) Version 6.00, part of FSL (FMRIB's Software Library, www.fmrib.ox.ac.uk/fsl). Analyses were performed using a mixed-effects model (FLAME1) and Z statistic images were thresholded using clusters determined by Z>2.58 and a (corrected) cluster significance threshold of P=0.05.*

Figure S5: Thumb activity – individual peaks

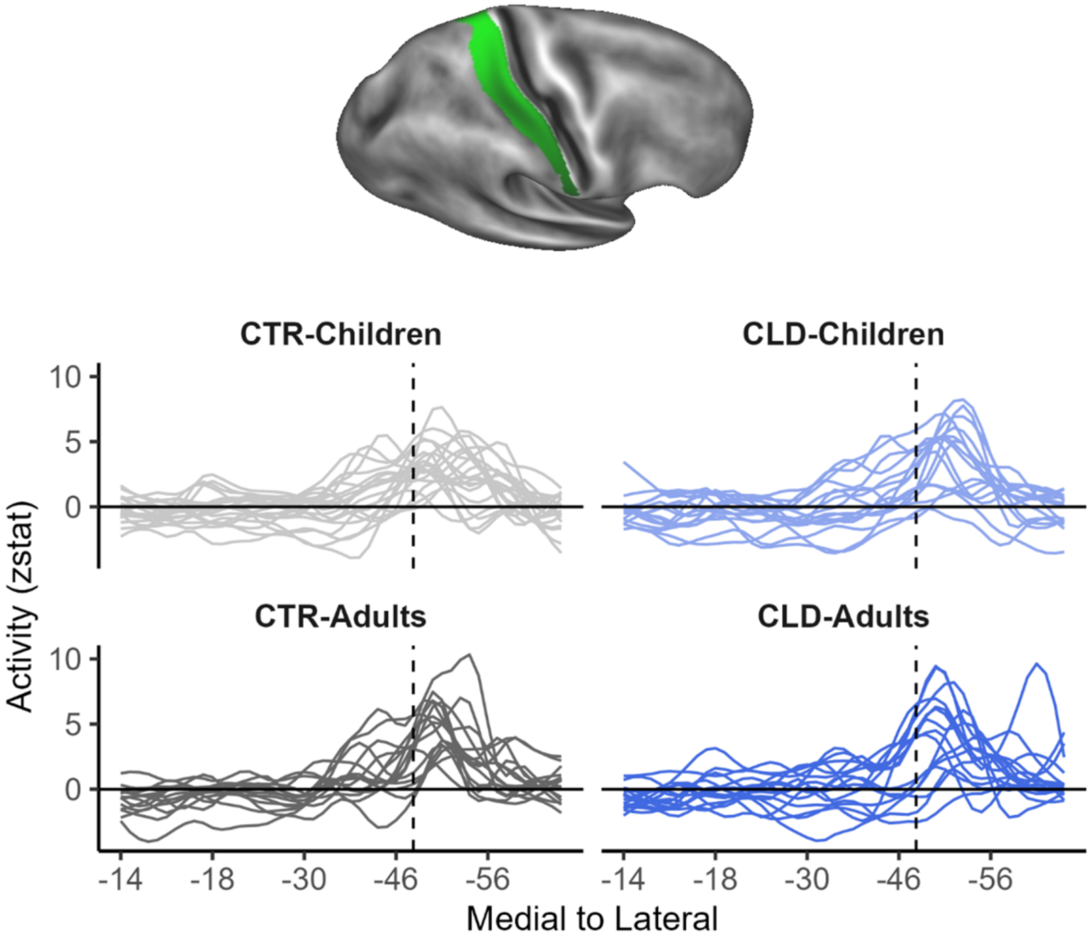

Figure S5. Individual activity profile for thumb stimulation. *Activity (z values) profile plots of all participants along the BA1-2 strip (highlighted in green) within the postcentral gyrus, in the hemisphere ipsilateral to the missing or non-dominant hand (i.e. contralateral to the stimulated thumb). Across all participants, a clear peak of neural activity is observed laterally to the centre of the hand area, which is marked by the vertical dashed line. This region corresponds to the centre of gravity for the five digits, as estimated by a meta-analysis[10]. Notably, in one of the CLD adults, the activity profile exhibits a double peak. These findings suggest consistent neural representation of the hand across participants. CLD children (N=16), CLD adults (N=16), CRT children (N=20), CRT adults (N=15). Source data are provided as a Source Data file.*

Figure S6: Group contrast – CLD > CRT, Body parts stimulation

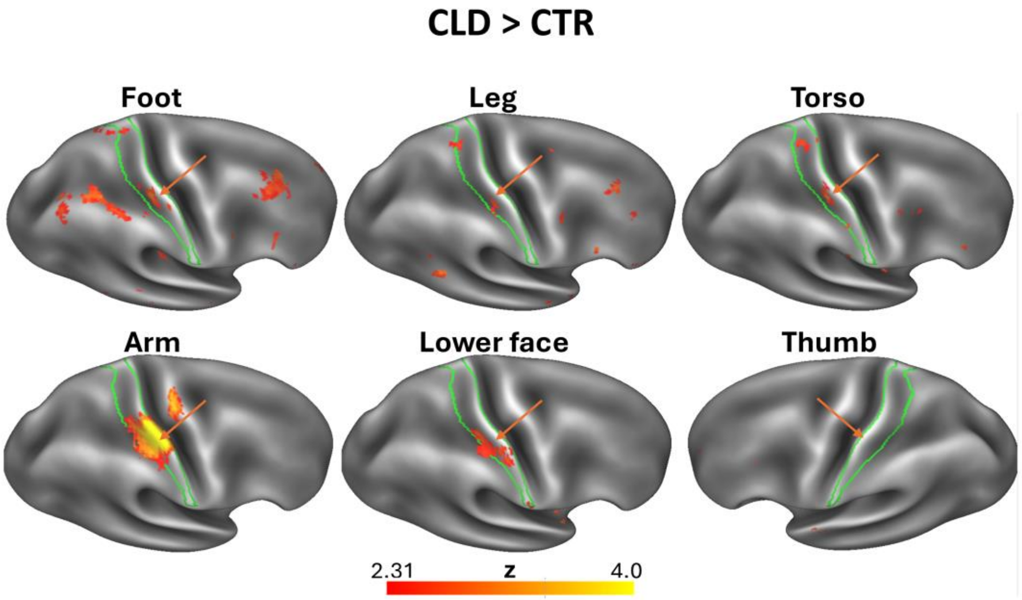

Figure S6. Differential activity (CLD > CRT) when each body part is stimulated. *Group-contrast (CLD > CTR) activation maps during stimulation of the residual/nondominant arm. Compared to control (CTR) participants, individuals in the CLD group demonstrated significantly greater activation in the contralateral missing-hand representation area. Data were analysed using a mixed-effects model (FLAME1). Following multiple-comparisons correction, only the arm region reached significance (Z-statistic thresholded at Z > 2.58, with a cluster-level corrected significance of P = 0.05). The remaining plots display Z-statistic maps thresholded at Z ≥ 2.31 (P < 0.01, uncorrected). The colour bar represents Z values.*

Figure S7: Activity profile within BA3b

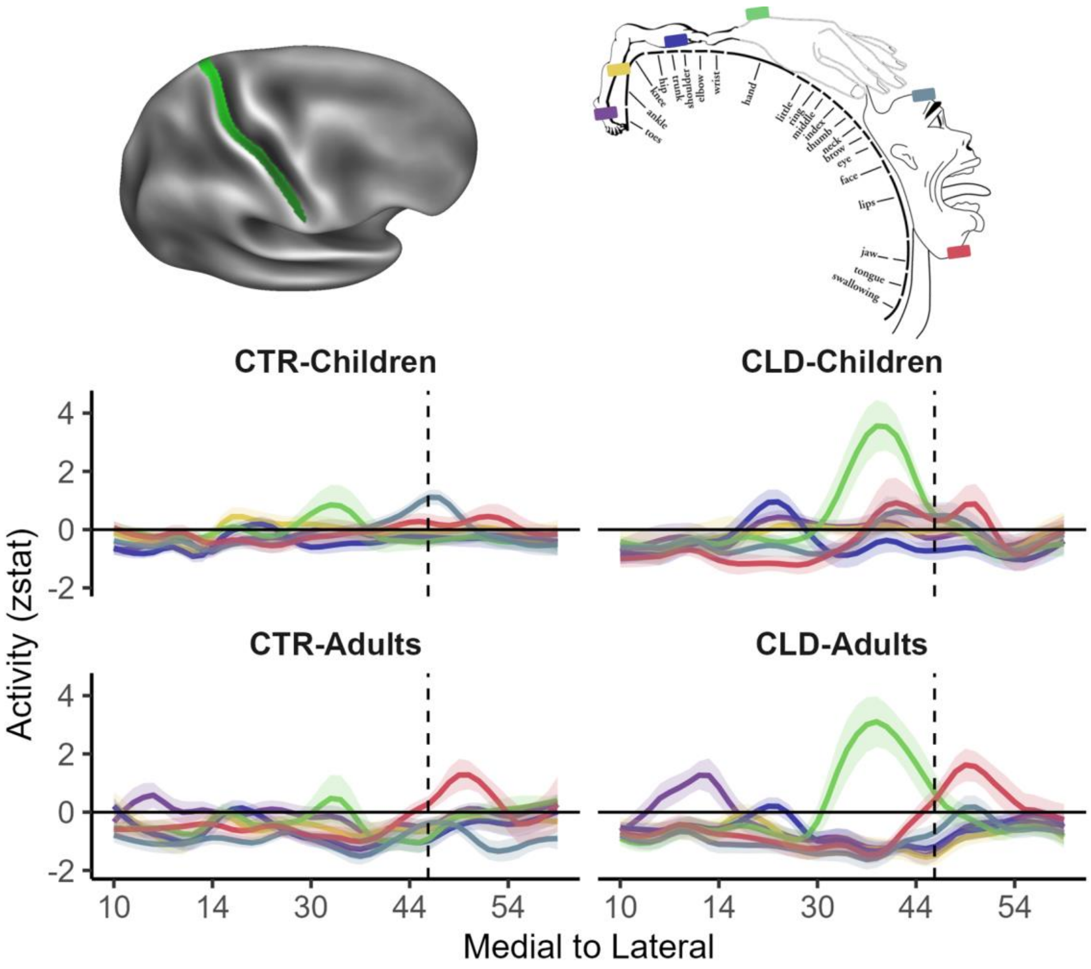

Figure S7. Global topography: descriptive plots showing remapping across the entire S1 homunculus (area 3b). *This plot displays the averaged activity (z-values) profiles for each group and body part along area 3b of S1, providing a comparison with the analyses presented in Figures 4 and 5 of the main manuscript, which focused on the more posterior areas of S1 (i.e., areas 1 and 2). The activity profiles observed in area 3b reveal both similarities and differences compared to those seen in areas BA1-2. Notably, the topographic organization appears more pronounced in this BA1-2 region, particularly for medial activities. For instance, stimulation of lower body parts (foot, leg, and torso) does not consistently activate area 3b, except in the case of the foot for CLD adults, and potentially CRT adults. Additionally, stimulation of the lower face elicits clear activity in area 3b, but predominantly in adults. All annotations and labels in this figure adhere to the same conventions used in Figure 4 for ease of comparison.*

Figure S8: Global S1-peak activity results.

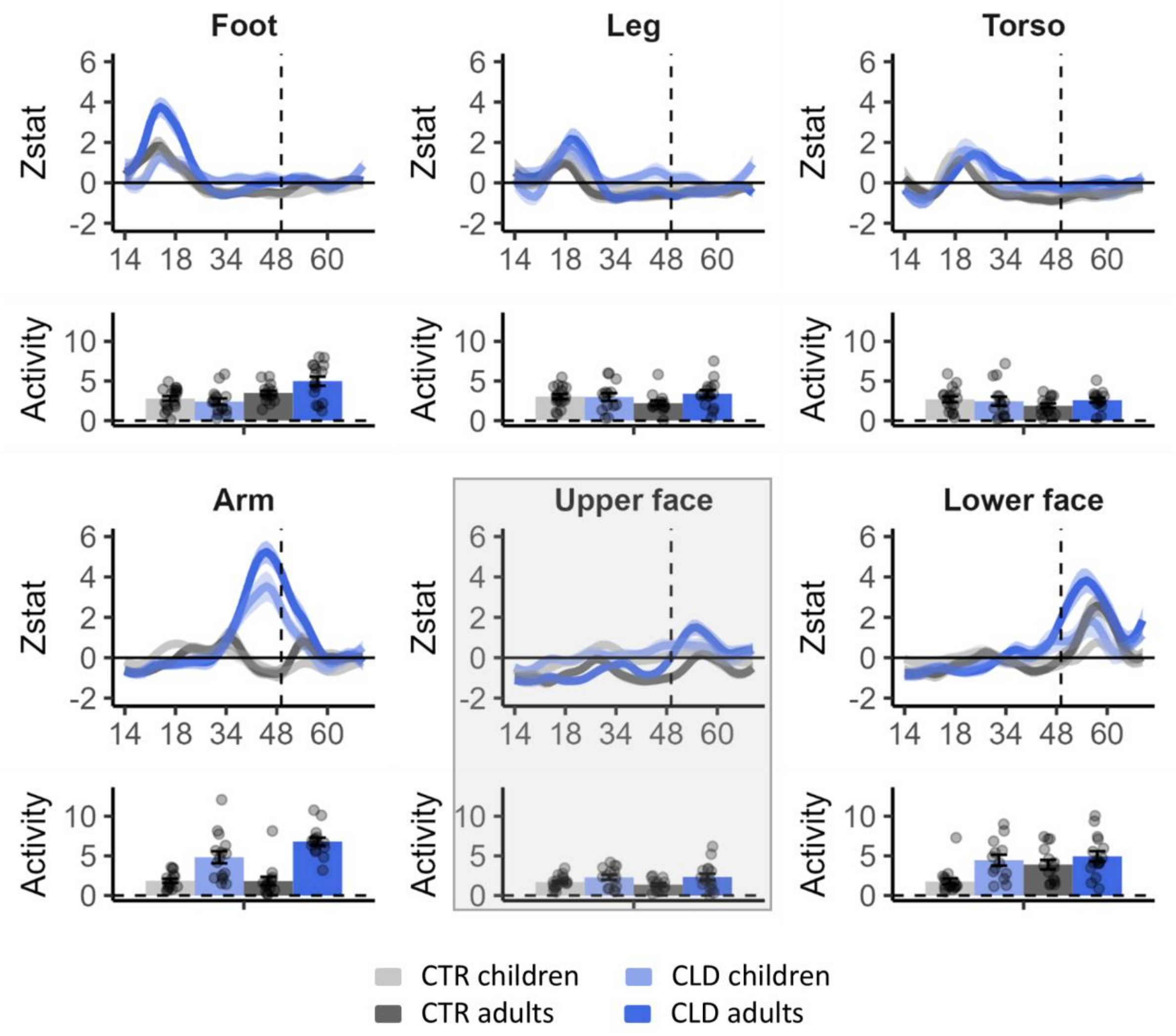

Figure S8. Averaged activity profiles and peak activities along S1. *Averaged activity (z values) profile plots (top panels) along S1 in response to stimulation of different body parts for each group, and the corresponding peak activity values of each individual and related group means (bottom panels). This analysis (detailed in the Supplementary Results section) complements the peak activity shift results presented in Figure 5 of the main manuscript. Notably, stronger activity in CLD participants was observed primarily in body parts adjacent to the deprived hand, specifically the residual arm and lower face, while the upper face remained unaffected. This localised pattern contrasts with the findings from the peak shift analysis (Figure 5), which demonstrated a more widespread impact of sensory deprivation. Error bars indicate standard error of the mean. All annotations and labels in this figure follow the same conventions as in Figure 5. CLD children (N=16), CLD adults (N=16), CRT children (N=20), CRT adults (N=15). Source data are provided as a Source Data file.*

Figure S9: Initial weights for the main model

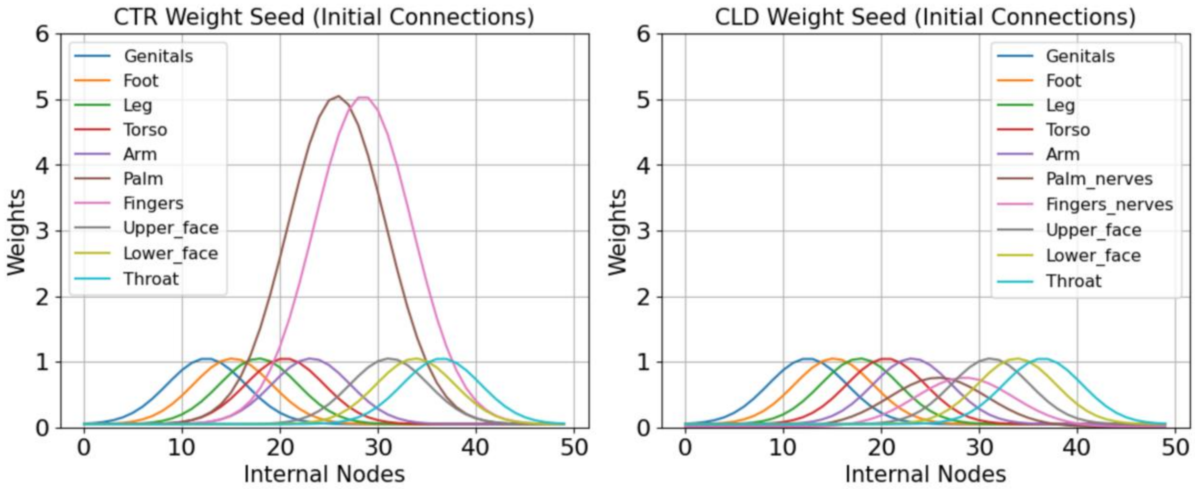

Figure S9. Initial connection weights between body parts and internal neurons (main model). *The figure shows how strongly individual body parts are connected to individual neurons. The left panel represents connections of the CTR group, while the right panel represents connections of the CLD group. For example, neuron 10 has connection with value ~0.9 with genitalia and connection with value ~0.2 with leg. Notice that for the CLD model (right), palm and fingers are replaced by the corresponding residual nerves and these connections are weaker than the connections of other body parts (see the Results section in the main manuscript). Except for the residual nerves, all bell-shaped curves are vertically shifted by 0.05. See the subsection Setting of Initial Connection Weights for connection parameter values and details.*

Figure S10: Model analysis – Higher frequency usage

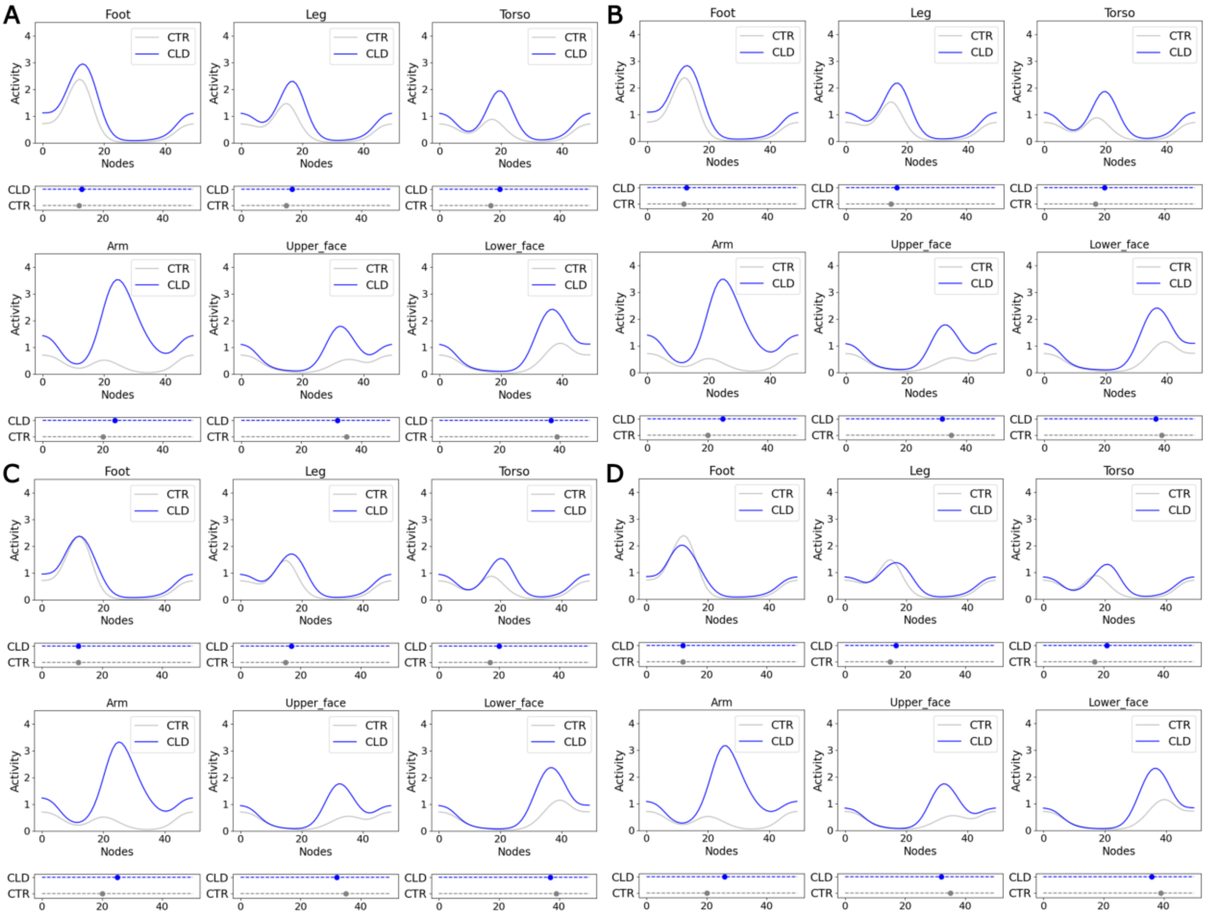

Figure S10. Effect of higher frequency usage of leg of CLDs on homunculus adaptation. *For CTR and CLD activations in subfigure A, baseline parameters are used. For CLD in subfigures B, C and D original stimulation frequency of leg was increased by 25%, 150% and 300%, respectively. Annotations as indicated in Figure 6 of the main manuscript.*

Figure S11: Model analysis – Lower frequency usage

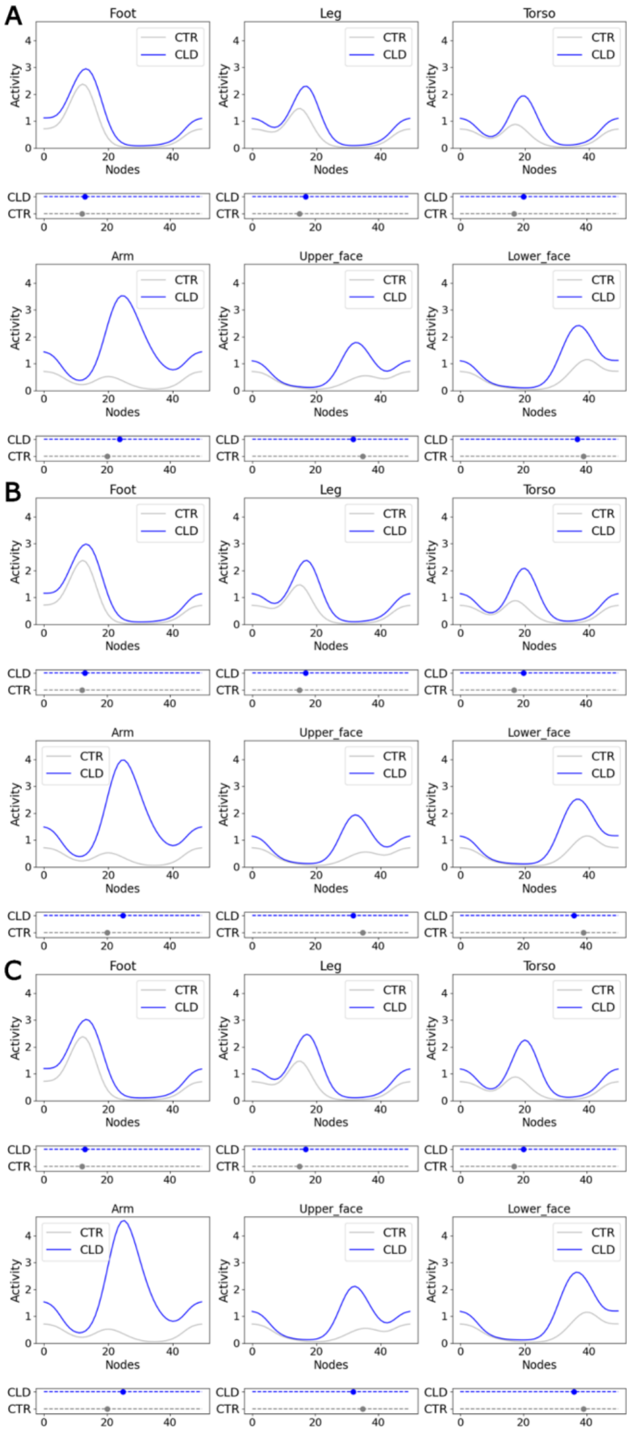

Figure S11. Effect of lower frequency usage of arm and hand nerves of CLDs on homunculus adaptation. *For CTR and CLD activations in subfigure A, baseline parameters are used. For CLD in subfigures B and C, original stimulation frequency of arm and hand residual nerves was decreased by 15% and 30%, respectively. Annotations as indicated in Figure 6 of the main manuscript.*

Figure S12: Initial weights for the rudimentary model

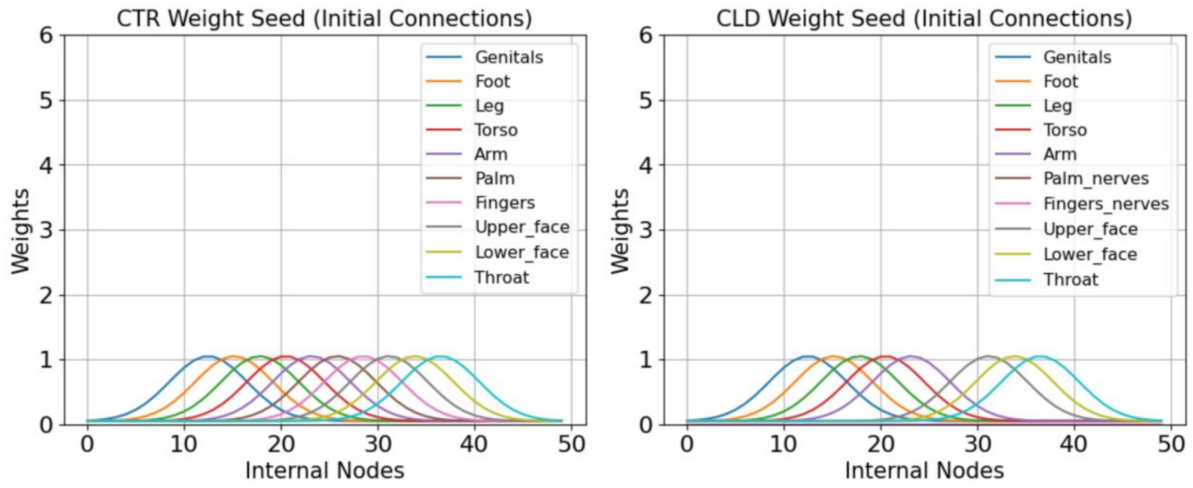

Figure S12. Initial connection weights between body parts and internal neurons (rudimentary model)*. (Left) Initial connection weights for CTR group. All body parts have same connection pattern. (Right) Initial connection weights for CLD group. Notice that Hand (i.e., Palm and Fingers) inputs are completely missing. Annotations as indicated in Figure S9. See the subsection Setting of Initial Connection Weights for connection parameter values and details.*

Figure S13: Rudimentary model results

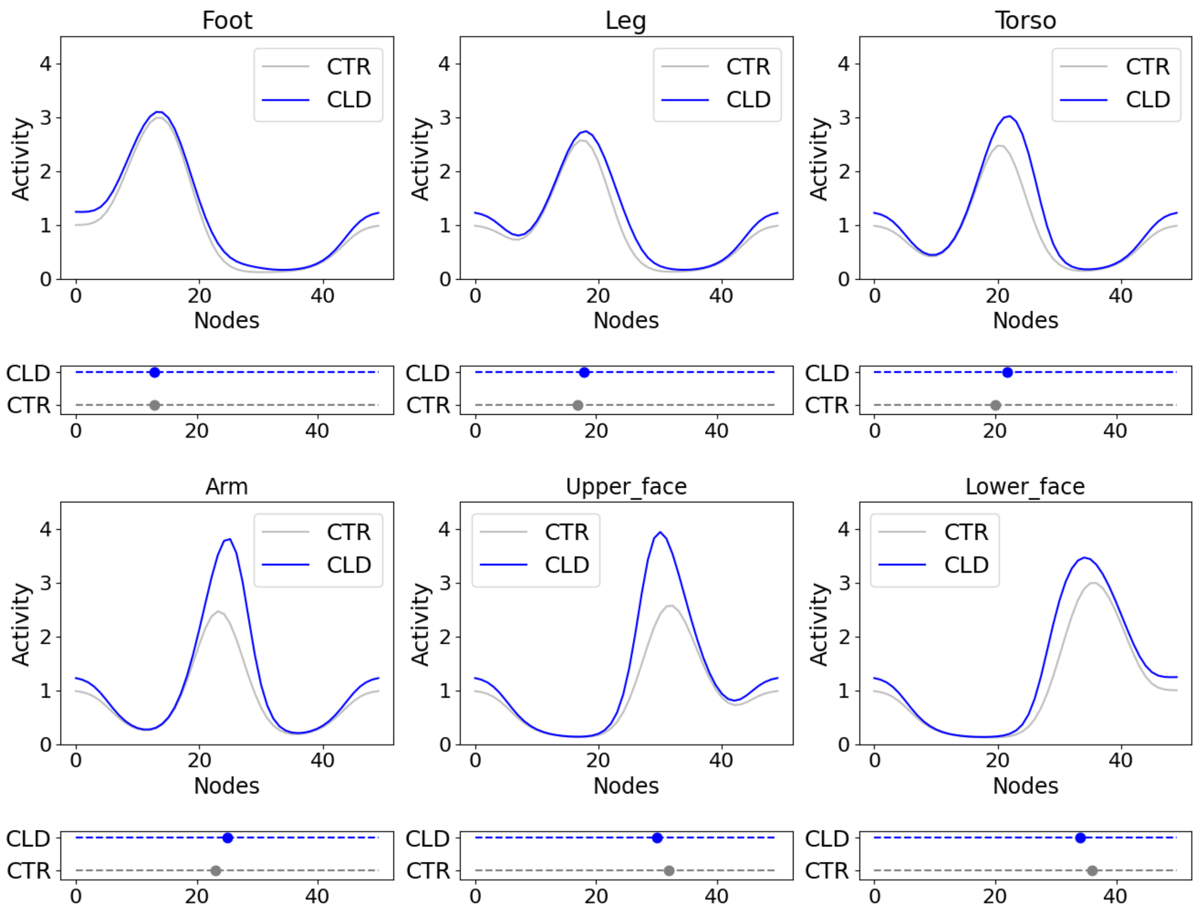

Figure S13. ***Peak position and activity plot of the rudimentary model.*** *See text for details. All annotations and labels in this figure follow the same conventions as in Figure 6.*

Figure S14: Local remapping beyond S1

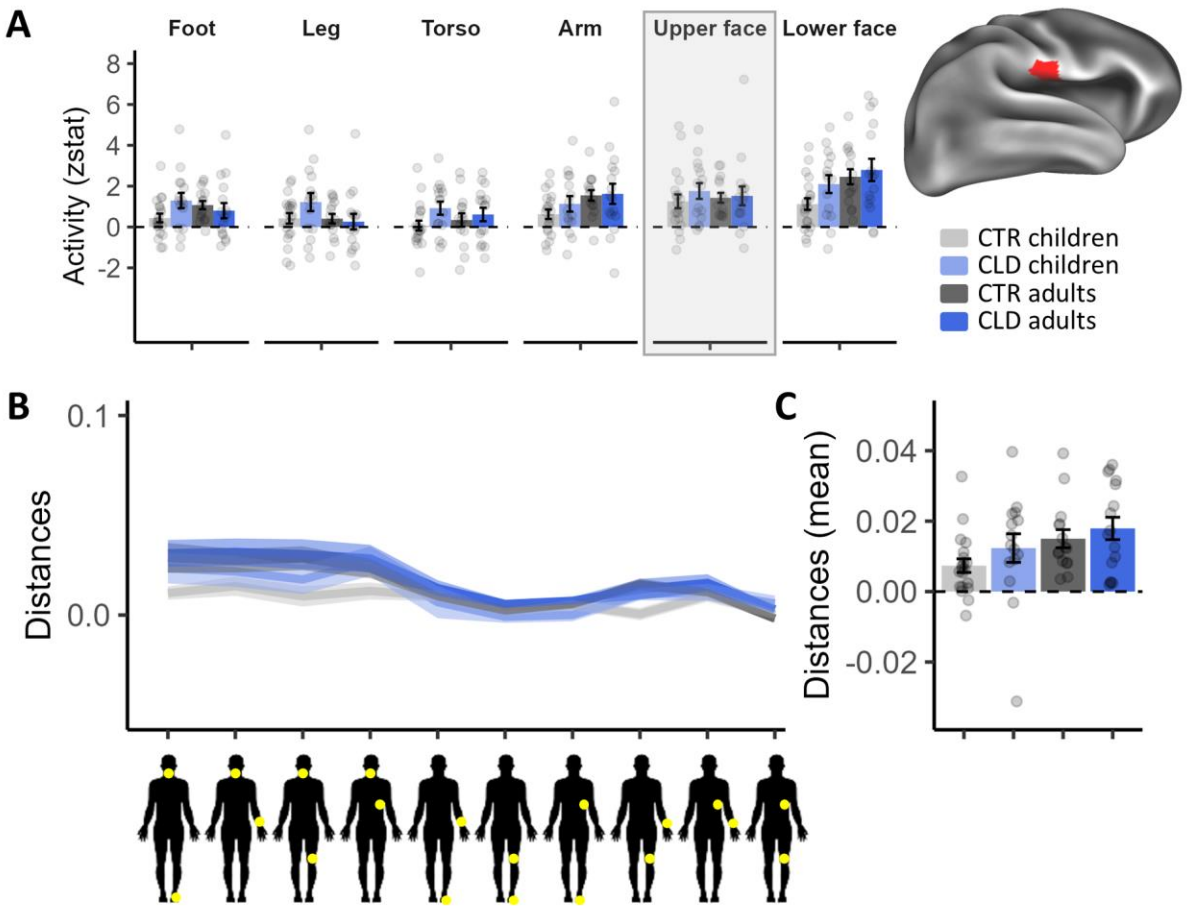

Figure S14. S2: Activity and Distances show no differential activity due to a congenital limb difference. *To investigate topographical changes beyond S1, we defined a ROI within S2 (Panel A, brain plot to the right) using the Op4 label from Glasser's multimodal parcellation[9] in the hemisphere contralateral to the missing/nondominant hand (in CLD/CTR, respectively).* ***(A)*** *Bars indicate z-stat activity within the S2 mask averaged across participants for each group and body part. We found no consistent effects of limb differences which were independent of age.* ***(B-C)*** *Line plot showing pairwise Euclidian distances (RSA analysis) for each group, which were averaged per group. We found a significant main effect for age and no main effect for limb difference nor an interaction. Error bars in panel A and C, and shaded areas in panel B indicate standard error of the mean. All annotations are as in Figure 3. CLD children (N=15), CLD adults (N=16), CRT children (N=20), CRT adults (N=15). Source data are provided as a Source Data file.*

Figure S15: Model analysis – hand frequency manipulation

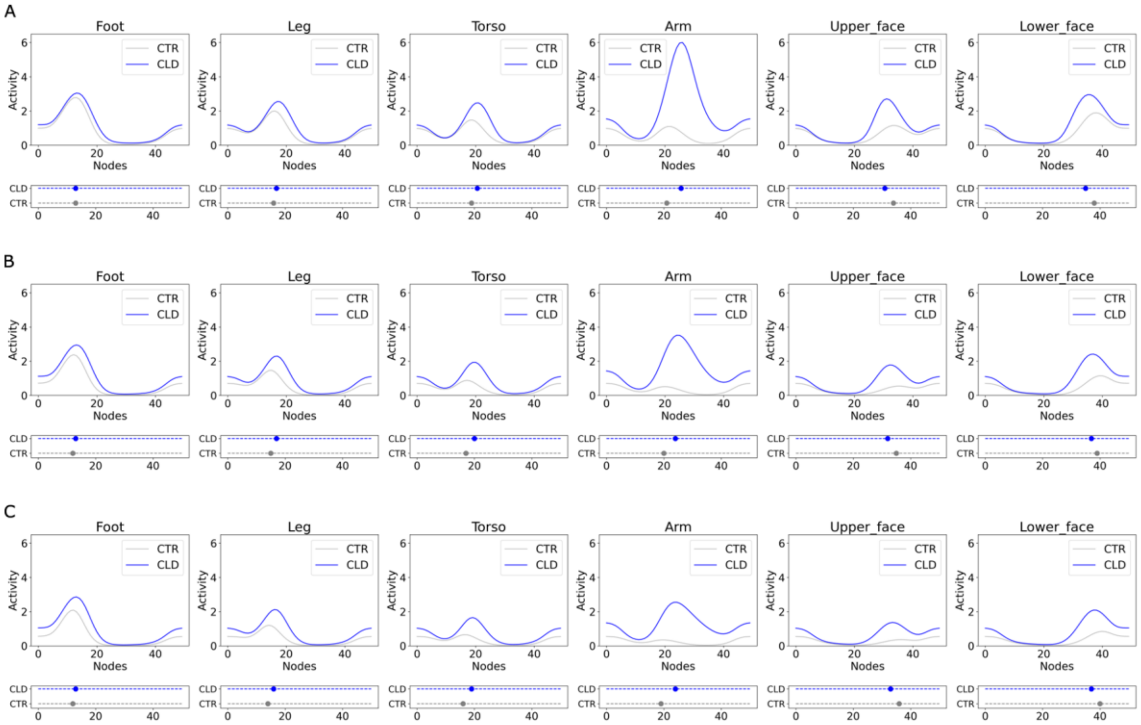

Figure S15. Effect of hand stimulation probability on model activations. *The hand was stimulated 1 (0.05, A), 3 (0.15, B), and 5 times (0.25, C) more frequently than other body parts. All other parameters were set to baseline values. See text for details. Annotations as indicated in Figure 6 of the main manuscript.*

Supplementary tables

Supplementary table 1: Behavioural sub-tasks and objects

1. **Padlock:** Put key in the padlock, twist, and remove from suitcase.
2. **Velcro:** Open a Velcro book bag (long Velcro strip).
3. **Plastic envelope:** Open a plastic envelope and remove plaster packet.
4. **Plaster:** Open plaster
5. **Buttons:** Do three buttons of varying sizes (large, medium, small)
6. **Jar:** Remove lid from a jar (unscrew)
7. **Pen:** Remove lid from a pen (pull)
8. **Lego:** Separate Lego bricks, two standard Lego bricks and two flat Lego bricks
9. **Tin:** Remove lid from a wide tin (pull)
10. **Nut and bolt:** Unscrew nut from a small wooden screw (traditional construction toy)
11. **Screwdriver:** Use a screwdriver to put a screw into a wooden block (traditional construction toy)
12. **Glove:** Place a glove on the intact hand
13. **Salad box:** Open a salad box
14. **Beads:** Thread three beads
15. **Sweet:** Open a twisty sweet

Linear mixed-models outputs (ANOVA tables)

Supplementary table 2: ANOVA output for co-use index

Terms	Sum Sq	Mean Sq	NumDF	DenDF	F value	Pr(>F)
Age	12.10	12.10	1	60.03	49.95	< 0.001***
Limb difference (LD)	36.90	36.90	1	60.04	152.36	< 0.001***
Age:LD	1.96	1.96	1	60.04	8.08	0.006**

Supplementary table 3: ANOVA output for proportion of use time

Terms	Sum Sq	Mean Sq	NumDF	DenDF	F value	Pr(>F)
Age	1.35	1.35	1	60.08	34.31	< 0.001***
Limb difference (LD)	2.68	2.68	1	60.09	67.89	< 0.001***
Body part (BP)	73.57	18.39	4	56.00	466.76	< 0.001***
Age:LD	0.25	0.25	1	60.08	6.28	0.015*
Age:BP	2.02	0.50	4	4,625.23	12.80	< 0.001***
BP:LD	24.67	6.17	4	4,625.23	156.50	< 0.001***
Age:LD:BP	1.08	0.27	4	4,625.23	6.83	< 0.001***

Supplementary table 4: ANOVA output for thumb peak position analysis

Terms	NumDF	DenDF	MSE	F	ges	Pr(>F)
Age	1	62	9.93	0.07	0.00	0.791
Limb difference (LD)	1	62	9.93	2.96	0.05	0.091#
Age:LD	1	62	9.93	0.00	0.00	0.992

Supplementary table 5: ANOVA output for thumb peak activity analysis

Terms	NumDF	DenDF	MSE	F	ges	Pr(>F)
Age	1	62	5.1	2.64	0.04	0.110
Limb difference (LD)	1	62	5.1	0.03	0.00	0.859
Age:LD	1	62	5.1	0.38	0.01	0.540

Supplementary table 6: ANOVA output for local remapping activity analysis in the deprived hand area

Terms	Sum Sq	Mean Sq	NumDF	DenDF	F value	Pr(>F)
Age	0.33	0.33	1	62	0.30	0.586
Limb difference (LD)	37.32	37.32	1	62	34.26	< 0.001***
Body part (BP)	6.39	1.60	4	0	1.47	1.000
Age:LD	0.94	0.94	1	62	0.86	0.357
Age:BP	16.26	4.07	4	248	3.73	0.006**
LD:BP	108.39	27.10	4	248	24.87	< 0.001***
Age:LD:BP	8.76	2.19	4	248	2.01	0.094#

Supplementary table 7: ANOVA output for local remapping distances analysis in deprived hand area

Terms	Sum Sq	Mean Sq	NumDF	DenDF	F value	Pr(>F)
Age	0.02	0.02	1	62	10.78	0.002**
Limb difference (LD)	0.04	0.04	1	62	28.33	< 0.001***
Age:LD	0.00	0.00	1	62	2.81	0.099#

Supplementary table 8: ANOVA output for global peak position analysis in S1

Terms	Sum Sq	Mean Sq	NumDF	DenDF	F value	Pr(>F)
Age	7.26	7.26	1	58.53	0.91	0.343
Limb difference (LD)	244.47	244.47	1	58.50	30.78	< 0.001***
Body part (BP)	21.52	5.38	4	218.43	0.68	0.608
Age:LD	31.54	31.54	1	58.49	3.97	0.051#
Age:BP	40.44	10.11	4	223.10	1.27	0.281
LD:BP	867.47	216.87	4	223.06	27.31	< 0.001***
Age:LD:BP	33.86	8.46	4	223.05	1.07	0.374

Supplementary table 9: ANOVA output for global peak activity analysis in S1

Terms	Sum Sq	Mean Sq	NumDF	DenDF	F value	Pr(>F)
Age	10.26	10.26	1	57.88	4.29	0.043*
Limb difference (LD)	51.15	51.15	1	57.86	21.38	< 0.001***
Body part (BP)	90.75	22.69	4	221.13	9.48	< 0.001***
Age:LD	4.11	4.11	1	57.86	1.72	0.195
Age:BP	47.49	11.87	4	220.99	4.96	< 0.001***
LD:BP	143.46	35.86	4	220.96	14.99	< 0.001***
Age:LD:BP	25.10	6.27	4	220.96	2.62	0.036*

Supplementary table 10: ANOVA output for local remapping activity analysis in S2

Terms	Sum Sq	Mean Sq	NumDF	DenDF	F value	Pr(>F)
Age	0.33	0.33	1	62	0.30	0.586
Limb difference (LD)	37.32	37.32	1	62	34.26	< 0.001***
Body part (BP)	6.39	1.60	4	0	1.47	1.000
Age:LD	0.94	0.94	1	62	0.86	0.357
Age:BP	16.26	4.07	4	248	3.73	0.006**
LD:BP	108.39	27.10	4	248	24.87	< 0.001***
Age:LD:BP	8.76	2.19	4	248	2.01	0.094#

Supplementary table 11: ANOVA output for local remapping distances analysis in S2

Terms	Sum Sq	Mean Sq	NumDF	DenDF	F value	Pr(>F)
Age	0	0	1	62	5.18	0.026*
Limb difference (LD)	0	0	1	62	1.87	0.177
Age:LD	0	0	1	62	0.12	0.725

Supplementary table 12: Double peaks and no peaks

Group	Reason	Body part	N
CTR Adults	Double peak	Arm	7
CTR Children	Double peak	Arm	2
CLD Adults	Double peak	Foot	1
CTR Adults	Double peak	Foot	1
CLD Children	Double peak	Leg	1
CTR Adults	Double peak	Leg	4
CTR Children	Double peak	Leg	1
CTR Adults	Double peak	Torso	3
CTR Children	No peak	Torso	2

Supplementary table 13: Model specifications

Supplementary table 13: *Summary of parameter differences between individual model scenarios. See Methods for details.*

BP*=body parts,* ***PR****=peripheral reorganization*

Model	Peripheral Reorganization of CLD	BP stimulation frequency	Initial connection patterns between the BP and nodes
PR + homeostasis	Yes	P=0.15 for palm and fingers and corresponding nerves; P=0.05 for other body parts	CTR: higher magnitude for hand and fingers, CLD: lower magnitude for palm and fingers residual nerves; Both: same magnitude for other BP (see Figure S9)
Rudimentary homeostasis	No	P=0.05 for all BP	No connections for palm and fingers for CLD, same magnitude for others (see Figure S12)
PR + homeostasis + behaviour (CLD leg 25% stimulation increase)	Yes	P=1.25*0.05=0.0625 for CLD leg; For others see "PR + homeostasis"	see "PR + homeostasis"
PR + homeostasis + behaviour (CLD leg 300% stimulation increase)	Yes	P=4*0.05=0.2 for CLD leg; For others see "PR + homeostasis"	see "PR + homeostasis"
PR + homeostasis + behaviour (arm)	Yes	0.7*0.15=0.105≤P≤0.15 for CLD palm and fingers nerves, 0.7*0.05=0.035≤P≤0.05 for CLD arm; For others see "PR + homeostasis"	see "PR + homeostasis"

Supplementary table 14: Model observations

Supplementary table 14. Alignment of the models with the key empirical observations Observation1-4 *(see Section "Key observations to be explained by the model" in the Methods section of the main text). Observation 1: Arm peaks difference CLD vs CTR; Observation 2: Activation peak shifts CLD vs CTR; Observation 3: Higher activation peaks magnitudes for CLD; Observation 4: Decreasing activation magnitudes.*

BP*=body parts,* ***PR****=peripheral reorganization (see Supplementary table 13 for summary of individual specifications)*

Model	Obs. 1	Obs. 2	Obs. 3	Obs. 4
PR + homeostasis	Yes	Yes	Yes	Yes
Rudimentary homeostasis	Yes	Yes for all BP except for foot	Yes	No
PR + homeostasis + behaviour (CLD leg 25% stimulation increase)	Yes	Yes	Yes	Yes
PR + homeostasis + behaviour (CLD leg 300 % stimulation increase)	Yes	Yes for all BP except for foot	No	Yes
PR + homeostasis + behaviour (arm)	Yes	Yes	Yes	Yes

Supplementary table 15: Group contrasts

Supplementary table 15. *MNI coordinates, p-value, z score and number of voxels for the group analysis shown in Figure S6 (CLD > CTR, arm stimulation). Analyses were performed using a mixed-effects model (FLAME1), with Z-statistic images thresholded at Z > 2.58 (one-sided) and a cluster significance threshold (corrected) of P = 0.05. COG: Centre of Gravity.*

	**Contrast**	**Voxels**	**P**	**MAX**				**COG**		
				z	x	y	z	x	y	z
CLD > CLD										
	Arm	494	<0.001	4.31	48	-24	56	47.6	-23.9	55.8

Supplementary References

1. Root, V. *et al.* Complex pattern of facial remapping in somatosensory cortex following congenital but not acquired hand loss. *Elife* **11**, 1–31 (2022).

2. Magosso, E., Zavaglia, M., Serino, A., di Pellegrino, G. & Ursino, M. Visuotactile Representation of Peripersonal Space: A Neural Network Study. *Neural Computation* **22**, 190–243 (2010).

3. Manabe, S. & Wetherald, R. T. Thermal Equilibrium of the Atmosphere with a Given Distribution of Relative Humidity. *Journal of the Atmospheric Sciences* **24**, 241–259 (1967).

4. Straka, Z., Noel, J. P. & Hoffmann, M. A normative model of peripersonal space encoding as performing impact prediction. *PLoS Comput Biol* **18**, 1–23 (2022).

5. Nevalainen, P., Lauronen, L. & Pihko, E. Development of human somatosensory cortical functions - What have we learned from magnetoencephalography: A review. *Frontiers in Human Neuroscience* **8**, 1–15 (2014).

6. Ruben, J. *et al.* Somatotopic organization of human secondary somatosensory cortex. *Cerebral cortex (New York, N.Y. : 1991)* **11**, 463–73 (2001).

7. Reed, C. L., Shoham, S. & Halgren, E. Neural Substrates of Tactile Object Recognition: An fMRI Study. *Human Brain Mapping* **21**, 236–246 (2004).

8. Gentile, G., Guterstam, A., Brozzoli, C. & Ehrsson, H. H. Disintegration of Multisensory Signals from the Real Hand Reduces Default Limb Self-Attribution: An fMRI Study. *Journal of Neuroscience* **33**, 13350–13366 (2013).

9. Glasser, M. F. *et al.* A multi-modal parcellation of human cerebral cortex. *Nature* **536**, 171–178 (2016).

10. Holmes, N. P. & Tamè, L. Locating primary somatosensory cortex in human brain stimulation studies: Systematic review and meta-analytic evidence. *J Neurophysiol* **121**, 152–162 (2019).
