# Supplementary material for: Global remapping of the sensory homunculus emerges early in childhood development
Source: Nat Commun. 2026 Feb 24;17:1591. doi: 10.1038/s41467-025-66539-5 (PMC12932738; doi:10.1038/s41467-025-66539-5)
Supplement: Supplementary file 2 — Reporting Summary [file 41467_2025_66539_MOESM2_ESM.pdf]

nature portfolio

Corresponding author(s): Raffaele Tucciarelli

Last updated by author(s): Sep 12, 2025

Reporting Summary

Nature Portfolio wishes to improve the reproducibility of the work that we publish. This form provides structure for consistency and transparency in reporting. For further information on Nature Portfolio policies, see our Editorial Policies and the Editorial Policy Checklist.

Statistics

For all statistical analyses, confirm that the following items are present in the figure legend, table legend, main text, or Methods section.

n/a	Confirmed	
☐	☒	The exact sample size (*n*) for each experimental group/condition, given as a discrete number and unit of measurement
☐	☒	A statement on whether measurements were taken from distinct samples or whether the same sample was measured repeatedly
☐	☒	The statistical test(s) used AND whether they are one- or two-sided<br>*Only common tests should be described solely by name; describe more complex techniques in the Methods section.*
☐	☒	A description of all covariates tested
☐	☒	A description of any assumptions or corrections, such as tests of normality and adjustment for multiple comparisons
☐	☒	A full description of the statistical parameters including central tendency (e.g. means) or other basic estimates (e.g. regression coefficient) AND variation (e.g. standard deviation) or associated estimates of uncertainty (e.g. confidence intervals)
☐	☒	For null hypothesis testing, the test statistic (e.g. *F*, *t*, *r*) with confidence intervals, effect sizes, degrees of freedom and *P* value noted<br>*Give P values as exact values whenever suitable.*
☒	☐	For Bayesian analysis, information on the choice of priors and Markov chain Monte Carlo settings
☒	☐	For hierarchical and complex designs, identification of the appropriate level for tests and full reporting of outcomes
☐	☒	Estimates of effect sizes (e.g. Cohen's *d*, Pearson's *r*), indicating how they were calculated

Our web collection on statistics for biologists contains articles on many of the points above.

Software and code

Policy information about availability of computer code

Data collection	Matlab R2020b (MathWorks) was used to run the script to control an MRI-compatible tactile device.
Data analysis	Statistical analyses were performed using custom scripts written in Matlab R2020b (MathWorks), R version 4.1.3 (R Core Team, 2022) with RStudio (2021.09.0 Build 351), and Python 3.10.6 with Spyder 5.3.3.<br>The code used to implement the computational model used in this work can be found here: https://colab.research.google.com/drive/1BGsSEKaZnzyG9paRM4KBtNazQ5WaVMt1?usp=sharing

For manuscripts utilizing custom algorithms or software that are central to the research but not yet described in published literature, software must be made available to editors and reviewers. We strongly encourage code deposition in a community repository (e.g. GitHub). See the Nature Portfolio guidelines for submitting code & software for further information.

Data

Policy information about availability of data

All manuscripts must include a data availability statement. This statement should provide the following information, where applicable:

- Accession codes, unique identifiers, or web links for publicly available datasets
- A description of any restrictions on data availability
- For clinical datasets or third party data, please ensure that the statement adheres to our policy

Data will be available in a repository (https://doi.org/10.17605/OSF.IO/NPZXB) or under request (r.tucciarelli@bbk.ac.uk)

April 2023

Research involving human participants, their data, or biological material

Policy information about studies with human participants or human data. See also policy information about sex, gender (identity/presentation), and sexual orientation and race, ethnicity and racism.

Reporting on sex and gender	Sex (either male or female) has been reported and determined based on self-report. Gender was not collected. Sex and gender were not considered as variables for analysis in this study, as they were not pertinent to the research objectives.
Reporting on race, ethnicity, or other socially relevant groupings	Race, ethnicity or other social grouping were not reported as not relevant for the study design.
Population characteristics	Our focus population comprised children aged 5 to 7 and adults aged 18 to 65 who have congenital limb malformations. The control group consisted of age-matched participants with typical development.
Recruitment	Participants were recruited through REACH (https://www.reach.org.uk/), a charity that supports children with limb difference and their families, and Opcare.
Ethics oversight	Recruitment was carried out in accordance with the University College of London committee (17205/001).

Note that full information on the approval of the study protocol must also be provided in the manuscript.

Field-specific reporting

Please select the one below that is the best fit for your research. If you are not sure, read the appropriate sections before making your selection.

☐ Life sciences ☒ Behavioural & social sciences ☐ Ecological, evolutionary & environmental sciences

For a reference copy of the document with all sections, see nature.com/documents/nr-reporting-summary-flat.pdf

Behavioural & social sciences study design

All studies must disclose on these points even when the disclosure is negative.

Study description	We employed a cross-sectional quantitative method, utilizing pediatric neuroimaging and behavioral analysis to examine cortical reorganization and adaptive behaviors in individuals with congenital limb differences (CLD).
Research sample	We tested 16 CLD children (mean age: 6.69 ± 0.60; 7 left-handed; 10 female) and 16 CLD adults (mean age: 41.19 ± 11.39; 5 left-handers; 12 female) with an isolated congenital upper limb malformation – of either the entire limb or the hand-plate.<br><br>For the control group, we tested 21 children (mean age: 6.71 ± 1.10; 4 left-handed; 13 female) and 16 adults (mean age: 39.56 ± 9.85; 5 left-handers; 7 female).<br><br>We were interested in studying deprivation- and use-dependent plasticity in the deprived primary somatosensory cortex and we hypothesised that altered behaviour during childhood is a driver for brain reorganisation observed in adulthood.
Sampling strategy	Participants were recruited through REACH (https://www.reach.org.uk/) and Opcare. Given that congenital limb malformation is a rare condition, we anticipated being able to collect data from approximately 15 to 20 children, accounting for potential dropouts.
Data collection	Data collection involved a object manipulation task, where participants were observed while interacting with various everyday objects, allowing for the assessment of compensatory behaviors in response to their congenital limb differences.<br><br>An fMRI experiment followed the behavioral session, where participants were exposed to a series of tactile stimulations delivered via soft pneumatic actuators (SPA) to body parts (feet, legs, torso, arm and lower face) known to be involved in compensatory behaviour.
Timing	Data collection started in August 2020 and ended on February 2024
Data exclusions	Pre-esablished criteria for exclusions were: CLD individuals with a single malformed but functional hand were excluded as 'too functional'; individuals with no functional hands were also excluded.<br>One CLD participant was excluded from the study due to having three residual fingers and a functional grip in the malformed limb.
Non-participation	Five participants (4 control children and 1 CLD adult) did not want to participate in the behavioural session<br>Three participants (1 control child, 1 CLD child and 1 control adult) did not want to participate in the fMRI experiment
Randomization	Participants were allocated into groups based on their congenital limb differences (CLD) and control status. Futhermore, participants were divided into children (5-7 years old) and adults (>25)

Reporting for specific materials, systems and methods

We require information from authors about some types of materials, experimental systems and methods used in many studies. Here, indicate whether each material, system or method listed is relevant to your study. If you are not sure if a list item applies to your research, read the appropriate section before selecting a response.

Materials & experimental systems

n/a	Involved in the study
☒	☐ Antibodies
☒	☐ Eukaryotic cell lines
☒	☐ Palaeontology and archaeology
☒	☐ Animals and other organisms
☒	☐ Clinical data
☒	☐ Dual use research of concern
☒	☐ Plants

Methods

n/a	Involved in the study
☒	☐ ChIP-seq
☒	☐ Flow cytometry
☐	☒ MRI-based neuroimaging

Plants

Seed stocks: N/A

Novel plant genotypes: N/A

Authentication: N/A

Magnetic resonance imaging

Experimental design

Design type: There was no specific task. Participants were required to watch videos while in the scanner and received tactile stimulation (block design)

Design specifications: The MRI session consisted of three functional runs, one structural run, and a fieldmap run. The fieldmap was acquired after the first functional run. The remaining two functional runs and the structural scan followed in that order. The protocol was flexible and could be adjusted based on each participant's needs.

Each run consisted of 5 block repetitions for each of the 7 tested body part conditions, resulting in a total of 15 block repetitions per body part across the three runs. All analysed participants completed the three runs. Tactile stimulation within each 9 sec block began 1 second after the block started to allow for pressure stabilization and lasted for 7.5 seconds, followed by 0.5 seconds of no stimulation before the next block began.

Each stimulation involved triplets of frequencies (5 Hz, 15 Hz, 30 Hz) repeated five times. The frequency changed every 400 milliseconds with a 100-millisecond gap between frequencies. The tactile blocks were interleaved with six 9 sec "null blocks" interspersed throughout the run, to allow for BOLD signal relaxation for baseline estimation.

The total run time was 401.6 seconds (277 volumes with a TR of 1.45 seconds).

Behavioral performance measures: No behavioural measures were taken in the scanner.

Acquisition

Imaging type(s): functional and structural

Field strength: 3T

Sequence & imaging parameters: Task fMRI data were acquired using a multiband GE echo planar imaging (EPI) sequence with an acceleration factor of 4. The field-of-view (FOV) consisted of 72 slices (TR: 1450 ms, TE: 35 ms, FA: 70°) with a spatial resolution of 2 mm isotropic.

A whole brain anatomical T1-weighted (MPRAGE) image was also collected with a 1 mm isotropic spatial resolution (FOV: 192×192×176, TR: 2530 ms, TE: 3.34 ms, FA: 7°, TI: 1100 ms, GRAPPA factor: 2).

Area of acquisition: whole brain

Diffusion MRI ☐ Used ☒ Not used

Preprocessing

Preprocessing software	All MRI data pre-processing and analysis was carried out using FMRIB Software Library (Jenkinson et al., 2012 FSL, versiob 6.0) as well as scrips written in MATLAB (version R2020b) and R (4.2.0) which were developed in-house.
Normalization	Functional images obtained at the second-level analysis were normalised to the MNI template using a non-linear transformation.
Normalization template	MNI152
Noise and artifact removal	Standard MRI and fMRI preprocessing was carried out using FSL tools. Estimated motion parameters were included in the GLM to identify brain regions functionally activated during tactile stimulation.
Volume censoring	Volumes with excessive motion or unusual signal intensity (frame-to-frame displacement larger than 0.9) were flagged for exclusion (using the FSL function fsl_motion_outliers) and included in the model as additional regressors of no interest (one for each outlier).

Statistical modeling & inference

Model type and settings	To localise brain regions activated during tactile stimulation, we used a voxel-based General Linear Model (GLM). For each participant, the three contrast images (one per run) of each body parts obtained from the first-level analysis were further processed with a second-level analysis using a fixed-effect model in FSL. These second-level contrast images were then processed with a group analysis using a random-effect model in FSL (FLAME 1) with the following parameters: Z threshold=2.58, cluster p threshold =0.05, cluster-corrected for multiple comparisons.<br><br>We also employed a representation similarity analysis (RSA) to quantify how distinct the information is for the different body parts within the S1 hand area. For each participant and run, we extracted the beta weights (representing brain activity) estimated with FEAT from each ROI. We then calculated the pairwise distance between these beta patterns across all body parts (excluding the thumb), using the cross-validated Mahalanobis distance (Walther et al., 2016). This distance reflects the dissimilarity between brain activity patterns evoked by different body parts.
Effect(s) tested	We were mainly interested in identifying brain regions active during tactile stimulation of body parts of interest (i.e., estimating the somatosensory body map). We therefore estimated BOLD responses relative to baseline (i.e., no stimulation).

Specify type of analysis: ☐ Whole brain ☐ ROI-based ☒ Both

Anatomical location(s)	We examined the global topography for the study's main body parts (feet, legs, torso, arm and lower face) along the full extent of S1 (BA1-2) in the deprived/non-dominant hemisphere.<br>We created a mask for the postcentral gyrus using the Glasser's multimodal parcellation encompassing BA1 and BA2 (Glasser et al., 2016). Since this combined ROI did not fully cover the medial aspect of the central sulcus, we also added region BA5m. This S1 ROI was then parcellated into 49 smaller sub-regions (i.e., bins) used for our main line and peak analyses (i.e., global S1 remapping).<br><br>For each participant, we also created hand-specific functionally-defined ROIs that were used for further analyses (i.e., RSA). Each participant's ROI was defined by finding the area in the brain that showed the most activity when the thumb was stimulated. This peak activity point was then mirrored to locate the corresponding area in the deprived/non-dominant hemisphere. Since the thumb defines the lateral boundary of the hand area, the hand ROI was defined by combining the peak bin with eight bins medial to it and one bin lateral to it, encompassing a total of ten bins.

Statistic type for inference<br>(See Eklund et al. 2016)	A group analysis was performed using a random-effect model in FSL (FLAME 1) with the following parameters: Z threshold=2.58, cluster p threshold =0.05, cluster-corrected for multiple comparisons.
Correction	We used a cluster-based approach to determine whether a cluster of voxels was significant (cluster p threshold =0.05).

Models & analysis

n/a	Involved in the study
☒	☐ Functional and/or effective connectivity
☒	☐ Graph analysis
☒	☐ Multivariate modeling or predictive analysis
